# Variation in the delivery of telephone advice by emergency medical services: a qualitative study in three services

**DOI:** 10.1136/bmjqs-2018-008330

**Published:** 2019-01-12

**Authors:** Rachel O'Hara, Lindsey Bishop-Edwards, Emma Knowles, Alicia O'Cathain

**Affiliations:** School of Health and Related Research, University of Sheffield, Sheffield, UK

**Keywords:** prehospital care, qualitative research, health services research

## Abstract

**Background:**

An emergency ambulance is not always the appropriate response for emergency medical service patients. Telephone advice aims to resolve low acuity calls over the phone, without sending an ambulance. In England, variation in rates of telephone advice and patient recontact between services raises concerns about inequities in care. To understand this variation, this study aimed to explore operational factors influencing the provision of telephone advice.

**Methods:**

This is a multimethod qualitative study in three emergency medical services in England with different rates of telephone advice and recontact. Non-participant observation (120 hours) involved 20 call handlers and 27 clinicians (eg, paramedics). Interviews were conducted with call handlers, clinicians and clinician managers (n=20).

**Results:**

Services varied in their views of the role of telephone advice, selection of their workforce, tasks clinicians were expected and permitted to do, and access to non-ambulance responses. Telephone advice was viewed either as an acceptable approach to managing demand or a way of managing risk. The workforce could be selected for their expertise or their inability to work ‘on-the-road’. Some services permitted proactive identification of calls for a lower priority response and provided access to a wider range of response options. The findings aligned with telephone advice rates for each service, particularly explaining why one service had lower rates.

**Conclusion:**

Some of the variation observed can be explained by operational differences between services and some of it by access to alternative response options in the wider urgent and emergency care system. The findings indicate scope for greater consistency in the delivery of telephone advice to ensure the widest range of options to meet the needs of different populations, regardless of geographical location.

## Background

Managing the ever-increasing demand for prehospital emergency care is a challenge for emergency medical services (also known as ambulance services) in many countries.^1–3^ As the first point of contact for patients seeking emergency care, emergency medical services can play a pivotal role in determining the most appropriate and timely care for patients within the wider urgent and emergency care system.^4^ For some patients an emergency ambulance may not always be appropriate, particularly the rising number of patients with low acuity problems and elderly patients with chronic health problems.^1 5^ Reassurance and practical preferences have been identified as valued aspects of care that might be addressed in ways other than sending an emergency ambulance.^6^

Consequently, in England, as in other countries, new models of prehospital emergency care and workforce roles have been developed to deliver quality care most appropriate to the clinical needs of patients.^7^ Telephone advice is one such model of care that aims to resolve low acuity calls over the phone, without sending an emergency ambulance. This generally involves clinician assessment following initial assessment by non-clinical call handlers and offers alternative response options, including self-care advice or referral to other services as appropriate. Clinician teams delivering assessment and advice can include both experienced generalists (paramedics, nurses) and specialists (mental health nurses, midwives). Telephone advice currently operates in a number of countries,^3^ and in England 6% of emergency calls during June 2018 were resolved over the telephone.^8^

Telephone advice can deliver the most appropriate and timely clinical response for some patients and increase the availability of an emergency ambulance for patients with life-threatening emergencies. Avoiding unnecessary emergency department attendances also has the potential to reduce crowding, a problem internationally.^9^ In England it is estimated that in 2015–2016, approximately 500 000 ambulance hours were lost due to turnaround delays at emergency departments.^10^ Diverting patients to more appropriate care elsewhere can reduce delays due to handover. A review of telephone advice concluded that, allowing for the limitations of small studies and system differences, clinician advice and disposition assigned were safe and appropriate.^3^ Telephone advice appears to be an acceptable response for patients based on the generally high levels of patient satisfaction.^11–13^

In England, 10 regional emergency medical services operate as part of the National Health Service and are accessible to the public via a 24-hour emergency telephone number (999). The operational structures of the 10 services are broadly the same, whereby emergency calls are assessed by non-clinical call handlers and prioritised for an ambulance response or telephone advice. Monthly Ambulance Quality Indicators for telephone advice and recontact with the ambulance service by these patients within 24 hours have always shown considerable variation between services in England.^8^ The most recent data for both indicators (July 2017) show the rates of telephone advice ranging from 5% to 20% and the rates for patient recontact within 24 hours ranging from 1% to 15%.^14^ The presence of service variation raises concerns about inequities in care,^15^ so it is important to understand potential contributory factors. A study of telephone advice by non-clinicians (using computerised decision support) for non-emergency healthcare conditions found that variation in patient disposition rates across five services in England appeared to be explained by operational differences.^16^ These operational factors are relevant to understanding variation in the delivery of telephone advice by emergency medical services, and include the tasks involved in delivering telephone advice, the workforce, the technology and the organisational context.

The aim of this study was to explore operational factors influencing the provision of telephone advice in three services reporting different rates of telephone advice and recontact.

## Methods

### Design

A pragmatic approach^17^ was adopted to conduct a multimethod qualitative study in three emergency medical services in England with varying rates of telephone advice and patient recontact. This involved non-participant observation and interviews with staff.

### Study settings

Ambulance Quality Indicators^8^ were used to identify three services with a marked variation in the rate of calls ending in telephone advice and subsequent recontact with that service. Patient recontact was used as a possible indicator that the call was not resolved satisfactorily in the first instance. This selection was made based on the pattern of variation over time prior to service selection in November 2014, which remained consistent throughout 2014. One service was selected because it had high rates of telephone advice and low rates of recontact (service C). One service was selected because it had low rates of telephone advice and high rates of recontact (service B). The final service was selected as a contrast, having medium rates for both indicators (service A). Data collection occurred sequentially, starting with service A and ending with service C (table 1).

There was a time delay between selection of sites and data collection to allow sufficient time for the research permissions process. At the time of data collection (November 2015–September 2016), some indicators had changed. For example, service A changed from having medium rates to having a high telephone advice rate and a low recontact rate (table 1).

**Table 1:** Ambulance Quality Indicator rates for telephone advice and recontact for participating services

	Service A	Service B	Service C
When service was selected	Medium telephone advice rate. Medium recontact.	Low telephone advice rate. High recontact.	High telephone advice rate. Low recontact.
During data collection	High telephone advice rate. Low recontact.	Low telephone advice rate. High recontact.	Medium telephone advice rate. Low recontact.

This pattern of variation during data collection remained the same until July 2017.^14^ More recently, changes to the quality indicator recording criteria have reduced the extent of variation for telephone advice to between 3% and 8% in June 2018 and recontact rates are no longer collected. The pattern of variation in the rates of telephone advice in the three participating services remains the same as during data collection.

The 10 ambulance services in England cover large geographical areas. Two of the services in this study comprised large urban centres and large rural areas, whereas the other service was predominantly urban. Each of the service catchment areas comprised populations that are ethnically and socioeconomically diverse. From the calls observed there were no obvious differences in the types of low acuity calls identified for clinician assessment.

### Participant characteristics

Participants were selected purposively^17^ based on their involvement in the provision of telephone advice. Table 2 shows staff roles for observation and interview participants per service. Non-clinical call handlers provide an initial assessment of emergency calls using a computerised decision support system that prioritises calls and allocates a corresponding response (eg, immediate ambulance). Non-emergency calls identified as not requiring an ambulance are passed to a team of clinicians (eg, paramedics, nurses) for further assessment and identification of the most appropriate response, which may include self-care advice, referral to other services or that an ambulance is needed. Clinical managers organise and support the team of clinicians delivering telephone advice, which can include identifying gaps in resources needed and using their clinical experience in responding to calls. It was originally intended to interview three to four call handlers and clinicians in each service, but following the observations it was felt two interviews with call handlers would be sufficient. As service A had recruited three call handlers, we conducted the three interviews. In services A and B it was difficult to recruit more than the minimum three clinicians requested within the study time scale.

**Table 2:** Staff roles for observation and interview participants

	Service A		Service B		Service C	
Staff role	Interview	Observation	Interview	Observation	Interview	Observation
Call handler	3	7	2	8	2	5
Clinician	3	8	3	7	4	12
Clinician manager	1	–	1	–	1	–

### Data collection

#### Observation

Non-participant observation is a way of understanding the complexity of healthcare that might otherwise be poorly understood^18^ to derive insights that could not be obtained by other methods.^19^ The observation of staff in their ‘natural’ work environment combined with interviews (see below) helped to gain an understanding of staff actions and decisions in relation to telephone advice. The actions of healthcare professionals have been examined previously using observation in similar settings.^16 20–22^

Preparation for data collection entailed a day of familiarisation in two services (A, B) in order to identify a general observation framework. A familiarisation visit in service C was deemed unnecessary as the researcher has already conducted multiple observations and the observation framework had been established. Five observation shifts of 8 hours on different days and times were conducted (table 3). In total, data collection entailed 120 hours of observation across the three services and involved 20 call handlers and 27 clinicians.

**Table 3:** Details of observation data collection months, days and times

Service/Observation visit	Month	Day	Time (8 hours)
A,1	November	Weekend	Day/Early
A, 2	January	Weekend	Night
A, 3	January	Weekday	Day/Early
A, 4	March	Weekday	Day/Early
A, 5	March	Weekday	Day/Early
B,1	January	Weekday	Day/Early
B, 2	February	Weekday	Day
B, 3	February	Weekday	Evening
B, 4	February	Weekend	Night
B, 5	March	Weekday	Day/Early
C, 1	April	Weekday	Night
C, 2	May	Weekday	Day/Early
C, 3	May	Weekend	Day/Early
C, 4	August	Weekday	Evening
C, 5	August	Weekday	Evening

Data collection was predominantly focused on clinicians. Some observed individuals were selected by managers, but many were opportunistic during the visit. All individuals who were observed were informed about the study, provided with study information leaflets and invited to complete a consent form. No individuals refused observation. Individual calls were the unit of observation, and data collected included how telephone advice calls were managed (eg, communication with callers and other services, staff interactions), verbal information from call handlers and clinicians dealing with calls (eg, ad hoc interviews questioning participants on how telephone advice is delivered and their role), and more general researcher observations and reflections throughout each visit (eg, impressions of the environment—level of demand, activity, mood). All information was recorded using handwritten notes.

#### Interviews

A small number of semistructured telephone interviews with call handlers, clinicians and clinician managers were undertaken to complement the observational data. Potential volunteers were recruited via managers, and the final numbers interviewed reflect the pragmatic nature of recruitment in each service rather than data saturation. All interviewees had private contact with the researcher and were informed about the study, provided with study information leaflets and invited to complete a consent form. All interviews were audio-recorded. The topic guide (box 1) was the same for all participants.

Box 1 **Interview topic guide**

- Involvement in telephone advice.
- Perceived role of telephone advice in the service.
- Factors affecting the provision of telephone advice in the service (eg, population, staff, organisational, locality, national).
- What works well to ensure appropriate telephone advice decisions.
- What does not work well to ensure appropriate telephone advice decisions.
- What would improve telephone advice.

The same researcher (LB-E) conducted all data collection and was blind to Ambulance Quality Indicator data for the services throughout all observations and interviews. LB-E has an MSc in Health Psychology and experience in qualitative healthcare research.

### Data analysis

Observation notes were transcribed into electronic format for analysis after each observation shift. Audio recordings of interviews were transcribed verbatim and checked for accuracy. The data were analysed using a framework approach^17^ supported by the NVivo qualitative data analysis software.^23^ The coding was undertaken by two researchers (LB-E coded the observations and ROH coded the interviews). The coding frames developed for both the observation and interview data were reviewed and discussed by the researchers, and the themes were refined through a number of iterations. Further analysis conducted by ROH combined the observation and interview data for each service. The emerging findings were discussed at various stages with the team and members of the patient and public involvement group. LB-E was blind to the Ambulance Quality Indicator data throughout the coding of observations. ROH was aware of the Ambulance Quality Indicator data throughout.

## Results

Five key themes were identified in relation to possible operational factors influencing variation in rates of telephone advice and recontact: an organisational view of telephone advice as either supporting demand management or managing risk; the workforce involved in the delivery of telephone advice; clinician role demands and scope; decision support systems for triage and access to information; and access to alternative response options for patients. Where interview quotes are presented, they are used to concisely articulate issues consistent with the observational data.

### Different organisational views of telephone advice as either supporting demand management or managing risk

There was a consensus of opinion among participants in the observations and interviews that sending an ambulance is not necessary for every call received and that limiting the use of emergency ambulances ensures that they are available for life-threatening emergencies. Clinicians across all services were conscious of their professional responsibility and the potential risk that telephone triage posed for them personally. They did not see themselves as merely managing demand for ambulances, but as providers of appropriate care. Sometimes clinicians decided that an ambulance response was needed, as observed in each service.

Each patient you hear, you are clinically responsible, you are not there just to divert ambulances. As an ultimate goal you are there to give that patient the most appropriate care each time. (Service C, Interviewee 62)

Clinicians in services A and C regarded their organisations’ rationale for telephone advice as more aligned with organisational performance and achieving targets for ambulance response times, that is, demand management.

I think the ambulance [service] have a different view, I think it’s relating to targets. (Service A, Interviewee 59)

This was particularly evident during observations in service A where clinicians proactively sought to identify calls for telephone advice to save ambulances. These clinicians also had to check on callers waiting for a delayed ambulance so were acutely aware of demand.

In service B clinicians were more focused on the potential risk associated with telephone advice and conscious of a more risk-averse organisational orientation. Risk management was viewed as the key consideration in delivering telephone advice, and clinicians in this service would more readily send an ambulance to a patient initially triaged to telephone advice.

As I say, we’re a unit that’s very risk-averse. We will send [an ambulance] where there is any query. (Service B, Interviewee 70)

### Workforce variation in the delivery of telephone advice: skill-mix, training and morale

The skill-mix of clinicians involved in telephone advice varied across the three services, with services A and C appearing to have a wider skill-mix. Service B only had paramedics in the clinician team, whereas services A and C also employed other clinical disciplines. Staff in each service found responding to mental health crises challenging, but only service C employed mental health specialists in telephone advice.

Clinician teams comprised a mix of paramedics who were unable to work ‘on-the-road’ for health reasons and those actively choosing the role, with the latter group viewing the role more favourably. Clinicians and managers in each service appeared to recognise the value of staff making a conscious choice to do telephone advice and required staff to apply for the role. Service C appeared to have a higher concentration of paramedics who had chosen to work in telephone advice and referred to a selection process that assesses skills for the unique demands of triaging patients over the telephone.

We can choose our staff now, it used to be, I think it used to be a bit of a ‘if you’re off sick you can come here and do it’, but actually it’s a bit more of a privilege […] we won’t accept just anyone now. (Service C, Interviewee 56)

Variation was reported in relation to initial and subsequent training. Service C appeared to place the greatest emphasis on paramedics remaining ‘fully operational’ and doing at least one shift per month ‘on-the-road’. In contrast, clinicians in services A and B spent a minimal or no amount of time ‘on-the-road’.

Staff morale also appeared to differ by service. Clinicians in service B talked of people “going nowhere,” whereas in service C telephone advice was referred to as a “career development” path. Some of the clinicians in service B were demotivated by the limited scope to use their clinical skills and discretion, linked to lower organisational tolerance of the risk associated with resolving calls over the phone. Workload was identified as a barrier to ongoing training in service A, affecting morale and the ability to work effectively. The following interviewee described the impact of increased demand since they started in the role.

…now it just seems to be solidly one call after the other, without any break in-between, […] a lot of the time it can be demoralising. (Service A, Interviewee 63)

### Variation in role demands and scope for clinicians delivering telephone advice

Each service operated differently in terms of the tasks undertaken alongside offering telephone advice. Additional roles included calls to check on patients when the ambulance had been delayed and to upgrade the call response if necessary (referred to as ‘welfare calls’ in services A and B), providing advice to ambulance crews and passing information to primary care services. Clinicians in services A and B undertook this range of tasks, whereas in service C the clinicians were dedicated to offering telephone advice. Clinicians in service A appeared to experience the greatest challenge in managing the various tasks and spoke negatively about the impact of ‘welfare calls’ on their ability to undertake telephone advice when emergency medical service call volumes were high. In service B, where the clinicians dealt with a lower volume of calls, staff experienced less role conflict.

…We allocate at least two, three or four people every day, every shift to purely be doing nothing but welfare calls, which means those three or four clinicians aren’t [offering telephone advice], they aren’t doing the job properly, they’re just literally ringing up saying sorry. (Service A, Interviewee 61)

The services also differed in permitting proactive downgrading of some call responses by clinicians. In services A and C clinicians could listen in to calls being assessed by call handlers and intercept calls that they could deal with straight away. This included non-critical calls that could be downgraded to a lower priority response. This freed up call handlers to deal with incoming calls and helped in managing demand for an immediate ambulance response. In contrast, the option to downgrade calls was not in use in service B. During the observations in this service, one of the clinicians expressed frustration at this change and commented that it was “totally appropriate” to assess and downgrade these calls.

### Variation in decision support systems for triage and access to information about alternative response options

The general process of call handler and clinician triage was broadly similar across the three services, although variation was apparent in relation to specific practices. In service B the triage software used by call handlers routinely provides self-referral advice for selected low priority calls. This potentially reduces the number of calls for clinician telephone advice but may not provide callers with the clinician reassurance they seek. For example:

…occasions where the patient’s, sort of said to me, but I need a paramedic here now, and I’ve turned round and said, well you’re actually talking to a paramedic. (Service A, Interviewee 59)

In each of the services, clinicians used different triage software to support their decision making, but it was not obvious what impact this had on their decision making. All clinicians had access to a national Directory of Services and local sources of information on available response options. However, frustration was apparent in all services regarding the time taken to access information and difficulties keeping directories up to date. Delays in accessing information and services increased the likelihood of having to send an ambulance.

### Variation in access to alternative response options in the wider network of urgent and emergency care services

Variation in access to telephone advice response options (eg, referral to primary care services, mental health services and special response teams) was identified. However, variation in the availability of services and access criteria between localities within each service appeared to be as significant. Primary care referrals to access doctors and district nurses were sometimes very time-consuming, particularly ‘out of hours’. Referral delays also increased the likelihood of ambulance dispatch, particularly when the patient had deteriorated.

So you ring the district nurse and explain what you’ve got, and sometimes you have ‘yes we know this patient but we’re short of staff tonight so we can’t attend to that’. (Service B, Interviewee 69)

One option in service C that was not observed in the other two services was a taxi to facilitate timely transport of patients assessed as not needing an ambulance but unable to self-transport to hospital. This option was used to free up ambulances to attend other patients, thus reducing the risk of a delayed response for time-critical emergencies:

Call back to young adult experiencing pain in leg, ongoing problem, 111 advised to call 999. […] Patient says he can’t get public transport or walk to hospital, clear desire for ambulance. Able to get into car and agrees to taxi. Hospital has an urgent care centre. (Service C, Observation 2)

### Summary of variation between services

A summary of variation between services is provided in table 4, along with the rates of telephone advice and recontact. It appears that services A and C are different from service B in having more factors that might facilitate higher rates of telephone advice. This is consistent with them having higher rates of telephone advice in the Ambulance Quality Indicators. However, it is not clear why service A has a higher rate than service C. Fewer factors were identified that might influence recontact rates, but it is possible that the services with a less risk-averse orientation may be delivering telephone advice in a way that reassures patients their needs are being met and an ambulance is not necessary.

**Table 4:** Summary of variation between services and Ambulance Quality Indicator rates for telephone advice (TA) and recontact (RC)*

Service A	Service B	Service C
TA=medium (high)	TA=low (low)	TA=high (medium)
RC=medium (low)	RC=high (high)	RC=low (low)
Perceived as demand management tool.	Perceived as risk management tool—risk aversion.	Perceived as demand management tool.
Varied skill-mix.	Limited skill-mix.	Varied skill-mix.
Lower morale.	Lower morale.	Higher morale.
Limited skill development.	Limited skill development.	More staff in role as career choice.
Engaged in other tasks.	Engaged in other tasks.	Focused on telephone advice.
Can downgrade some calls.	Cannot downgrade calls.	Can downgrade some calls.
Higher workload.	Lower workload.	Higher workload.
More response options.	Fewer response options.	More response options.
Different triage software.	Different triage software.	Different triage software.

*Rate when service was selected (rate at data collection).

## Discussion

### Summary of findings

The findings appear to reflect different organisational orientations towards the perceived risk in resolving calls without face-to-face assessment, either acknowledging it as an acceptable approach for managing demand or adopting a more cautious risk-averse approach. Clinician skill variation concerned the extent of proactive selection and development of suitably skilled staff rather than allocating staff for health reasons. Role demands varied in relation to the tasks staff were expected and permitted to do, with extremes of high and low workloads impacting on the use of clinical skills and staff morale. Variability in access to suitable response options either facilitated or constrained the ability of clinicians to resolve calls with telephone advice. These findings aligned with telephone advice rates in each service, particularly explaining why one service had low rates.

### Findings in the context of relevant research

Telephone advice relies on organisations ensuring an appropriately skilled workforce to resolve calls over the phone, addressing emotional as well as medical needs of patients,^6^ but this was subject to variation. This is also an aspect of telephone advice that has been identified as needing more research to determine the level of skills and education required and the best decision support strategies to ensure consistent, appropriate and safe care.^3^

The impact of workload variation on staff activity and morale resonates with a survey of nurses delivering telephone advice for non-emergency healthcare conditions, where one-fifth of participants identified problems with training, workload and monotony as challenging for staff retention.^24^ Consideration of these organisational-level issues is therefore relevant to the retention of skilled staff in emergency medical services. Workforce planning may also be subject to influences beyond the organisation;. For example, in England, concerns have been raised about skill gaps in the clinical workforce needed to move away from hospital to community-based care for at-risk populations, including elderly patients with complex health needs.^25^

Eastwood *et al*’s review^3^ concluded that the effectiveness of telephone advice is limited by the range of options available to clinicians and suggests that the availability of alternative options may also reduce the level of recontacts. National healthcare policy in England advocates unrestricted referral rights for emergency medical service clinicians to other services in the wider urgent and emergency care network, including social care.^7^ However, it appears that there is still some way to go in achieving this given the inconsistency in availability and access to services observed in this and other studies in England.^10 26^

Overall, these findings appear consistent with the study of telephone advice by non-clinicians (using computerised decision support) for non-emergency healthcare conditions, where organisational differences in how services operated appeared to explain variation in patient disposition rates.^16^ Both studies highlight that telephone advice is a complex activity and delivery varies across services when it might have been assumed there would be greater standardisation of delivery within a national health service.

### Implications

Telephone advice offers potential benefits for the delivery of appropriate, timely and acceptable patient care, but operational variation between services may impact on equity of access to services and health outcomes.^15^ Rates of telephone advice have always shown considerable variation between emergency medical services in England, and the findings here suggest that some of this variation can be explained by operational differences between services, including motivation to undertake telephone advice, workforce skill and the nature of the work. The study also identified factors influencing variation in telephone advice that are not easily addressed by individual services, including access to alternative response options in the wider urgent and emergency care system. The variation identified implies scope for greater consistency in how telephone advice operates across services and the wider urgent and emergency care system to ensure the widest range of options to meet the needs of different populations, regardless of their geographical location. Efforts to address variation in the areas identified here also need to consider how to achieve operational consistency while retaining the ability to accommodate variation in the needs of specific populations.

### Strengths and limitations

A key strength of this study was the use of non-participant observation to explore the processes involved in delivering telephone advice. The observation of staff in their ‘natural’ work environment combined with semistructured interviews enabled us to gain an understanding of the complexity of telephone advice and organisational variation in three different services. While there have been a number of studies using observation to explore the actions of healthcare professionals in similar settings,^16 20–22^ there is a lack of research specifically examining variation in emergency medical service telephone advice across multiple organisations. Eastwood *et al* 3 identified small-scale studies and variation in telephone advice as limitations in their review. This study permitted the comparison of practices across three organisations with recorded variation in the amount of telephone advice and recontacts. The same researcher conducted all data collection, with regular review and feedback from members of the study team. Data were presented to the study team at various stages, including public and patient representatives.

In terms of limitations, this was a relatively small-scale study comprising a fixed number of observations and interviews to understand a complex activity. While this provided a considerable amount of qualitative data, it does to some extent represent a ‘snapshot’ of what was happening on the particular occasions that the services were observed. The study attempted to capture variation across times of the day and days of the week, but there may have been activity that was not captured. The consecutive nature of observational data collection across the three services increased the potential for seasonal bias; efforts to mitigate this included avoiding expected periods of unusual demand (eg, adverse weather, December, local events). Participants were the staff involved in front-line service delivery, either self-selected or invited by their manager, and although a range of perspectives were obtained we acknowledge that these do not necessarily reflect all staff views. National Ambulance Quality Indicator data were used to identify services with variation in the amount of telephone advice and recontacts. However concerns have been raised about the consistency of data reporting for calls resolved over the phone,^10^ and changes to the reporting criteria appear to have reduced the variation in rates of telephone advice.^8^ Finally, ROH was aware of the Ambulance Quality Indicator data when analysing the interviews, which could have introduced bias.

## Conclusion

The aim of this study was to explore operational factors influencing the provision of telephone advice in three services reporting different rates of telephone advice and recontact. The findings provide insights on the complexity of the process and key areas of variation across three services in England. Some of the variation appears modifiable, indicating scope for greater consistency in service delivery.
